# Modern Aspects of Burn Injury Immunopathogenesis and Prognostic Immunobiochemical Markers (Mini-Review)

**DOI:** 10.3390/biotech11020018

**Published:** 2022-05-27

**Authors:** Tatyana A. Kuznetsova, Boris G. Andryukov, Natalia N. Besednova

**Affiliations:** 1 Somov Institute of Epidemiology and Microbiology, Russian Federal Service for Surveillance on Consumer Rights Protection and Human Wellbeing, 690087 Vladivostok, Russia; andrukov_bg@mail.ru (B.G.A.); besednoff_lev@mail.ru (N.N.B.); 2 School of Medicine, Far Eastern Federal University (FEFU), 690091 Vladivostok, Russia

**Keywords:** burn injury, immunopathogenesis, innate and adaptive immunity, biomarkers

## Abstract

Burn injuries are among the most common peacetime injuries, with mortality ranging from 2.3% to 3.6%. At the same time, 85–90% of patients with burns are people of working age and children. Burn injury leads to metabolic disorders and systemic inflammatory response, inefficient energy consumption, and other physiological changes that can lead to dysfunction of organs and systems. The most formidable complication of burn injuries is sepsis mediated by multiple organ failure, the most common cause of poor prognosis in patients and has specific differences in these injuries. The purpose of this article was to dwell in detail on the most promising immunobiochemical markers of sepsis in the format of a mini-review, based on the main aspects of the immunopathogenesis of this complication. The pathogenesis of a burn injury and any general pathological process is based on an inflammatory reaction and large-scale changes in the skin and mucous membranes. This review is devoted to the progress in understanding the main aspects of the immunopathogenesis of burn lesions and the features of post-burn immune dysfunction, manifested by disorders in the innate and adaptive immunity systems. Attention is focused on the role in the immunopathogenesis of the development of systemic and local disorders in burn injury. Characterization of primary immunobiochemical markers of burn injury (cytokines, growth factors, C-reactive protein, procalcitonin, presepsin, matrix metalloproteinases, reactive oxygen species, nitric oxide, hemostasis parameters) is presented. The problem of treating burn lesions is associated with constant monitoring of the condition of patients and regular monitoring of specific immunobiochemical markers predicting sepsis for the timely initiation of a specific therapy.

## 1. Introduction

Burn injury is one of the most common types of injuries in peacetime. Mortality from burns in general ranges from 2.3% to 3.6%. At the same time, 85–90% are working-aged people and children. Of the surviving patients, a significant proportion need long-term medical, social, labor, and psychological rehabilitation [1,2,3]. Burns are among the most common traumatic injuries globally, representing a global public health problem. According to the WHO report for 2018, about 11 million cases of burns occur worldwide every year while burn injuries cause approximately 180,000 deaths worldwide [1]. Two-thirds of all cases of burn injuries occur in everyday life. Burns received as a result of military operations are of high relevance. An increase in the group of injured persons with severe and extremely severe lesions is apparent. Thus, more than 500,000 people seek medical care, approximately 40,000 require hospitalization, and 4000 deaths are recorded per year in the United States [1].

There are several types of burn injuries: chemical (acid and alkaline), electrical, and thermal, which are the most common. Small burns cause superficial damage, mediating only local skin reactions. However, with prolonged and extensive heat damage, burns can be extensive and cause systemic pathological reactions in patients [2,3].

A thermal (burn) wound (injuries) is an open injury or destruction of the skin, appendages, or mucous membranes. The severity, the nature of pathological changes, and the prognosis of burn injuries mainly depend on the depth and the localization of burn wounds and their area, the age and general condition of the organism, and several other factors matter. Extensive burns (severe burn injury or burn disease) are one of the most life-threatening injuries with high mortality [2,4]. Burn disease is a specific pathological process that develops after a thermal injury and is accompanied by damage to all self-regulating body systems. The body’s response is manifested by neuro-reflex, neuroendocrine, and inflammatory systemic reactions. Burn disease develops in a pronounced form, with superficial burns > 25–30% of total body surface area (TBSA) or deep burns > 10% TBSA [3,5].

**Figure 1:**
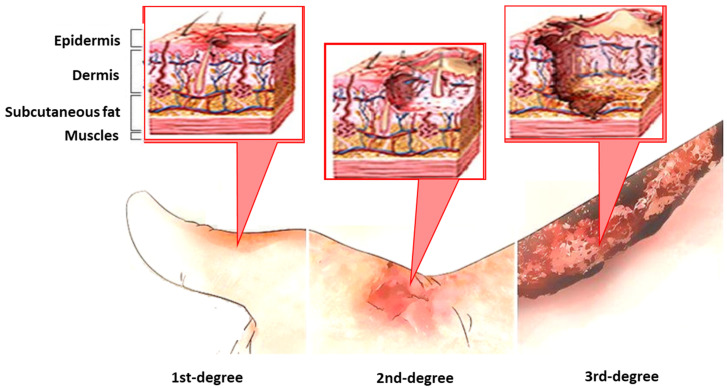
The classification of burns by degree is based on the depth of damage to the skin and other tissues. 1st-degree burns involve only the outer layer of skin, the epidermis; 2nd-degree burns involve the epidermis and some or all of the dermis; and 3rd-degree involve the entire dermis and destroy the hair follicles and sweat glands.

The risk of infection, especially nosocomial infection, is significant for patients with burn injury. The burn wound is fertile for colonization by endogenous and exogenous origin microorganisms. It is believed that infectious complications cause up to 75% of all deaths after thermal wounds. There is a risk of both local infection of the burn wound and the development of sepsis in this case. Sepsis (systemic inflammatory response syndrome, SIRS) is one of the most common causes of death in burn patients [4,6]. According to the recommendations published in 2016 and highlighted at the 45th Critical Care Society Congress of the Society of Intensive Care Medicine (SCCM), sepsis is defined as life-threatening organ dysfunction caused by a dysregulated host response to infection. This new definition emphasizes the primacy of the nonhomeostatic host response to infection, the potential lethality that is considerably more than a straightforward infection, and the need for urgent recognition [7]. Even a modest degree of organ dysfunction when an infection is first suspected is associated with in-hospital mortality of over 10% [5]. The main difference of burn sepsis is an increased risk of infection due to the loss of the skin as the first line of defense against microbial invasion. Sepsis rarely occurs during the first week; it more often develops several weeks or even months after a burn injury [6] (Figure 1).

Severe burn injury leads to significant disturbances in the hemostasis system, resulting in the development of coagulopathy and disseminated intravascular coagulation (DIC syndrome) [7]. Coagulopathy in burn patients is defined as a developing dysfunction of the hemostasis system, which is characterized by activation of procoagulant pathways, increased fibrinolytic activity, and a decrease in natural anticoagulants, and is considered as a risk factor for increased mortality both in the early and later periods after burn injury [7]. DIC syndrome is a complex and multifaceted disease characterized by activation of coagulation and fibrinolysis pathways, consumption of coagulation factors, and depletion of coagulation factors. DIC develops in patients with critically ill burns or patients with severe burns (up to 3 degrees) and extensive damage > 10% TBSA [8,9]. Disorders of the hemostasis system play a significant role in microcirculation disorders in severe thermal injury and can also be one of the causes of multiple organ dysfunction syndrome (MODS) [7].

Considering the above, there is a great need to identify and monitor the development of complications, including severe complications such as sepsis and septic shock, coagulopathy, and DIC syndrome, in patients with burns. The authors of our mini-review are doctors of laboratory medicine and clinical immunologists, who themselves repeatedly faced the choice of diagnostic tactics when faced with the question of clinicians: sepsis or not sepsis? Therefore, our manuscript is primarily addressed to combustiologists, who often ask this question.

This review aims to reveal the modern features of the immunopathogenesis of burn injuries and post-burn immune dysfunction and to determine the primary immunobiochemical markers of burn injuries and burn sepsis (cytokines, growth factors, C-reactive protein, procalcitonin, presepsin, matrix metalloproteinases, reactive oxygen species, nitric oxide, parameters of hemostasis).

## 2. Methods

Literature searches were conducted on PubMed, Web of Science, and EMBASE (through 13 February 2022). Search criteria used were: (burn injury OR thermal injury OR burn AND (contact thermal stimulation OR thermal injury OR burn injury OR localized hyperthermia OR thermode) AND (immunopathogenesis OR prognostic markers OR immunobiochemical markers OR predictor markers OR sepsis OR pathogenesis). Unpublished articles and reviews were not considered. Authors (BGA, TAK) individually selected all identified articles based on title and abstract. In the case of disagreement between the authors regarding the relevance of the article, the senior author (NNB) made the final decision Subsequently, the relevant articles were reviewed in full text to determine their final eligibility.

When analyzing the data, special attention was paid to identifying and monitoring the development of complications, including such severe ones as sepsis and septic shock, coagulopathy, and DIC, in patients with burns. The purpose of this review is to identify the modern features of the immunopathogenesis of burn lesions and post-burn immune dysfunction, to determine the primary immunobiochemical markers of burn lesions and burn sepsis.

As a result, 512 sources were found. Next, we searched for data on specific biomarkers of burn injury (cytokines, growth factors, C-reactive protein, procalcitonin, presepsin, matrix metalloproteinases, reactive oxygen species, nitric oxide, parameters of hemostasis) and their role in the immunopathogenesis of burn disease. Authors analyzed 258 articles, of which 61 were selected for inclusion in this review. The included publications contained data on the role of biomarkers such as cytokines, growth factors, C-reactive protein, procalcitonin, presepsin, matrix metalloproteinases, reactive oxygen species, and nitric oxide; the parameters of hemostasis in burn disease and sepsis complications; and studies that have shown their prognostic role for the diagnosis and therapy of this pathology.

## 3. Results and Discussion

### Modern Aspects of the Burn Injury and Burn Sepsis Immunopathogenesis

The basis of the immunopathogenesis of burn injury and any general pathological process is an inflammatory reaction, ultimately aimed at restoring the structure and function of the damaged tissue. A feature of the inflammatory response in burn injury is the scale of the skin and mucous membranes alteration. This barrier is damaged first in burns patients. The intensity of the inflammatory reaction depends on the type of burn, its depth, and the extent of the burned area. The cumulative injury of a thermal burn causes a severe inflammatory response, which leads to impaired immune function and vice versa. Immune system dysfunction caused by a burn injury exacerbates the inflammatory response. It can lead to a systemic inflammatory response (hyperinflammatory) or sepsis and increase mortality risk [10].

Dysfunction of the immune system in burn injuries is manifested by disorders in the innate and adaptive immunity systems. These disorders occur already in the initial post-burn period and are recorded for a long time. Post-burn immune dysfunction is a hallmark of critically ill burn patients. In general, immune dysfunction in severe burn injury and burn sepsis is characterized by a systemic (hyperinflammatory) immune response and immunosuppression. While the former causes tissue damage, the latter predisposes to infections [11,12].

Numerous experimental and clinical studies indicate that burn injuries are associated with immunosuppression [13,14,15,16,17,18]. Innate immune cells (monocytes/macrophages, neutrophils, dendritic cells, natural killer (NK), natural killer T cells (NKT)) are among the first to react to burns. Liver cells (hepatocytes, liver stellate cells, Kupffer cells, bile duct epithelial cells) participate. The reaction of immunocompetent and liver cells is expressed in the synthesis of cytokines, chemokines, adipocytokines, and other mediators (catecholamines, cortisol, reactive oxygen species (ROS)), mediating both a local inflammatory response and a systemic inflammatory process [16,17]. Considerable evidence of a disturbance of the functional activity of monocytes/macrophages, neutrophils, and NK cells is presented, and the role of these cells in the development of post-burn immune dysfunction has been shown in experimental and clinical studies [16,19,20].

Dysfunction in the system of adaptive immunity from the first days after thermal in-jury is manifested by a decrease in the total content of T-lymphocytes, with a decrease in the content of CD3+, CD4+, and CD8+ T-lymphocytes in the peripheral blood and a disorder of their functional activity [13]. As for the subpopulation of lymphocytes, several studies reflect the role of CD3+ CD4+ T-helpers, including Th-1 (T-helpers type 1) and Th-2 (T-helpers type 2) and Th17 (T-helpers type 17), CD3+ CD8+ T cells, and NK and NKT cells in burn injury and sepsis. In particular, it was shown that a decrease in the total number of T-lymphocytes in severe burn injuries was revealed due to the subpopulation of T-helper type 2 (Th-2) [13,16].

Researchers have recently paid particular attention to the role of subpopulations of γδ T cells, Th17, and Treg cells (regulatory T cells) in the immunopathogenesis of burns. The subpopulation of γδ T cells, which belongs to the cells of innate immunity, predominates in the skin and epithelial tissues. It is assumed that γδ T cells have different immunopathogenetic significance for burn injury. On the one hand, they contribute to increased survival, as evidenced by the increased mortality observed in mice γδ T cells that are deficient. However, on the other hand, γδ T cells are associated with subsequent immune dysfunction and damage to peripheral organs. In general, it is believed that the activation of γδ T cells after a burn is a protective function due to their participation in immune surveillance, tissue repair, and wound healing. Thermal injury patients with severe systemic inflammatory responses have an increased circulating γδ T cells concentration [17,18].

Regarding Th-17, there is growing evidence supporting the role of these cells in the immune response after a burn. These cells are involved in local and systemic immune responses as early as 3 h after a burn injury. Evidence shows increased IL-17 and IL-22 by these cells at the burn site and in the systemic circulation [21]. Th-17 cells have been shown to protect against local and systemic post-burn infections [21].

Hanschen et al. [22] evidenced the damaging effect of burn injury on Treg cells. The authors of another work resolved the issue of the involvement of Tregs in sepsis-induced immune paralysis [23]. They showed that elevated Tregs-produced cytokines and activation markers might play an essential role in the pathogenesis of sepsis and mortality of burned patients [23].

Thus, the basis of the pathogenesis of burn injury and any general pathological process is an inflammatory reaction. Dysfunction of the immune system in thermal injuries is manifested by disorders in the innate and adaptive immunity systems. All stages in the development of inflammation and the immune response are regulated by cytokines, which mediate both the local and systemic inflammatory processes. The balance of cytokines and other mediators plays a decisive role in regulating wounds’ initiation, progression, and resolution. A disbalance between pro- and anti-inflammatory systems can lead to either destructive hyperinflammation or paralysis of the immune system and the development of sepsis after burn injury [12].

**Figure 2:**
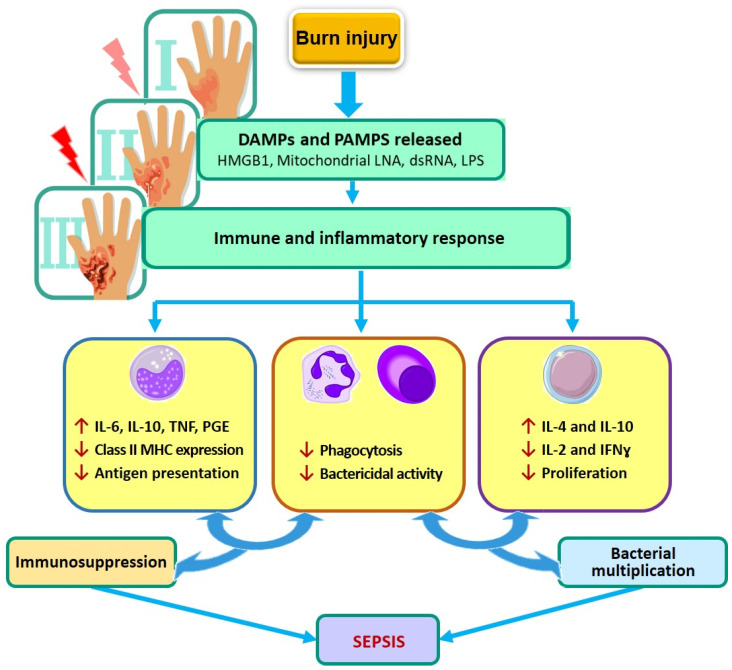
The scheme of post severe burn injury and burn sepsis pathogenesis. *Note*: DAMPs—damage-associated molecular patterns, PAMPs—pathogen-associated molecular pattern molecules, NK—natural killer, IL-2—interleukin 2, IFN-γ—interferon γ, Th-1—helper T lymphocyte 1, Th-2—helper T lymphocyte 2, MODS—multiple organ dysfunction syndrome (Figure by authors).

Figure 2 shows the scheme of post-severe burn injury and burns sepsis pathogenesis.

A comprehensive study of the burn injury and burn sepsis immunopathogenesis is an urgent task, the solution of which, at the present level, is necessary to prevent complications due to an abnormal immune response, and to develop methods for effective treatment of burns, including methods for modulating the protective potential of the macroorganism. The most crucial aspect for predicting the response to a burn injury, the likelihood of complications, assessing the processes of wound healing, and, ultimately, individual treatment of each patient is the monitoring of several immunobiochemical parameters (markers).

## 4. Immunobiochemical Markers of Burn Injury and Burn Sepsis

Numerous biologically active substances are involved in the immunopathogenesis of the development of systemic and local disorders in burn injury. Up to 200 biomarkers associated with burn injury have been identified. There are systemic and local biomarkers of burn injury. An evaluation of both is essential for understanding wound healing mechanisms and predicting the severity of burn injury patients and the therapy’s adequacy. These include biomarkers associated with metabolism, inflammation, and wound healing [24,25,26,27].

Taking into account immunopathogenesis, among the most important (key) immunobiochemical markers (or sensors) are:

- Cytokines and growth factors;
- Acute-phase proteins;
- Matrix metalloproteinases;
- Reactive oxygen species;
- Nitric oxide;
- Parameters of the hemostasis system [13,28,29,30,31,32,33,34,35,36,37].

These and other vital biomarkers are presented in Table 1.

### 4.1. Cytokines

The most critical biomarkers of burn injuries include cytokines due to their significant role in immunopathogenesis. An important role belongs to both proinflammatory (IL-1α, IL-1β, IL-6, IL-8, IL-12, TNFα, IFNγ, etc.) and anti-inflammatory (IL-4, IL-10, etc.) cytokines in the development of burn inflammation. The proinflammatory cytokines are involved in the mechanisms of chronization of the burn wound, having a significant impact on the course and outcome of inflammatory-reparative processes. The function of anti-inflammatory cytokines is to inhibit the excess synthesis of the central proinflammatory cytokines, which contributes to the restriction of the area of damage. Increased pro-inflammatory cytokines (TNF-α, IL-1, IL-6, etc.) prolong the inflammatory phase, leading to the chronization of burn wounds or hypertrophic scars [27].

Extensive burn injuries accompanied by an increased inflammatory response are characterized by an imbalance between proinflammatory and anti-inflammatory cytokines. The magnitude and dynamics of changes in proinflammatory cytokines reflect the severity of the burn disease and the nature of the healing of the burn. Most researchers note a significant increase in the level of these cytokines in the blood serum immediately after the burn and a decrease by 2–3 weeks [13,26,28,29]. According to other authors, elevated serum levels of pro- and anti-inflammatory cytokines were observed after the severe burn injury within 6–8 months, indicating sepsis complications [30].

These data suggest that cytokines have great potential for predicting burn disease outcomes and early diagnosis of burn sepsis.

### 4.2. Growth Factors

Growth factors play an essential role in the tissue’s reparation and wound healing, affecting cell migration, proliferation, and angiogenesis. An increase in serum hepatocyte growth factor (HGF) has been reported, promoting wound healing and angiogenesis stimulation in burned pediatric patients [31].

Moreover, an increased level of growth factor (TGFα), which affects cell migration, cellular proliferation, and angiogenesis [32], epidermal growth factor (EGF) [33], and fibroblast growth factor (BFGF) [32] has been noted. As for the proinflammatory colony-stimulating factors G-CSF and GM-CSF, elevated serum levels persisted for three years after severe burn injury [30].

### 4.3. Acute-Phase Proteins

Protein C or C-reactive protein is one of the central components of the acute phase, the generally recognized “gold marker” of inflammatory processes, which correlates with the severity of the inflammatory process and is a highly sensitive indicator of tissue damage. C-reactive protein is an important prognostic marker and an early predictor of sepsis in patients with severe thermal burns [34,35]. The burn disease is accompanied by dysproteinemia due to hypoalbuminemia, an increase in the blood levels of acute-phase proteins (CRP and fibrinogen) [36].

According to Jeschke, on the second day after severe burn injury, CRP values were observed in patients with a fatal outcome compared with survivors. These authors also found that if a patient had CRP values greater than 20 mg/dL on days 11–16 after burn injury, there was a 50% chance the patient would die [34]. The elevated levels of CRP were observed for a long time (up to 6 months), and with an increase in the area of burns, high levels were also observed [34].

Important biomarkers of systemic inflammation initiated by infection in burn sepsis are the blood levels of procalcitonin (PCT) and presepsin. PCT is a polypeptide that is an inactive precursor of calcitonin. The main inducers of its synthesis are endotoxins of Gram-negative bacteria, and TNF-α and IL-6. Among a large array of laboratory tests, PCT emerged as the leading biomarker to indicate the presence of systemic infection accurately and time-effectively. There is no single diagnostic value of PCT indicating sepsis development. However, as most studies follow, a value above 1.0 ng/mL is taken as a guide [37,38].

Several authors have testified to an increased level of PCT in burn sepsis [32,38,39,40]. So, Lavrentieva et al. [39] found that the PCT cut-off level of 1.5 ng/mL had the highest sensitivity (82%) and specificity (91.2%) in septic patients with severe burn injury. Mokline et al. [40] found that five days after burn injury, the PCT serum concentration was significantly different between infected and non-infected patients (5.44 ± 6.23 and 0.41 ± 0.64 ng/mL, respectively). The authors concluded that PCT levels correlate closely with sepsis severity, could have a prognostic value in the outcome, and repeated measurements were more useful than single values [40].

Presepsin is a circulating protein that increases rapidly in the blood during systemic infections, sepsis, severe sepsis, and septic shock. It was first described at the beginning of the 20th century in Japan. Further international multicenter studies have shown that:

(1) The mechanism of increasing PSP levels is fundamentally different from the mechanism of increasing proinflammatory markers such as TNF-α, IL-6, IL10, PCT, and CRP;
(2) During the induction of systemic inflammation, the increase in PSP occurs earlier and faster than the increase in other markers of sepsis [41].

Presepsin is normally present in very low concentrations in the serum of healthy individuals. In response to bacterial infections, its concentration increases within 2 h, according to the severity of the disease. The cut-off levels for sepsis have been reported between 400 and 600 pg/mL; its concentration significantly correlates with the severity of sepsis syndrome and in-hospital mortality [42].

In burn patients, a higher presepsin cut-off may be used to test for the presence of bacteremia [38].

Therefore, procalcitonin and presepsin are the important biomarkers of systemic inflammation initiated by infection in burn sepsis. Egea-Guerrero et al. [43] believe that the role of PCT in identifying infectious processes in critically burned patients is superior to CRP.

### 4.4. Proteases: Matrix Metalloproteinases

Proteases are enzymes responsible for the hydrolysis of proteins, primarily catalyzing the degradation reactions of the extracellular matrix. Matrix metalloproteinases (MMPs) and their inhibitors (tissue inhibitors of metalloproteinases—TIMPs), also called acute-phase proteins, play a fundamental role in extracellular matrix remodeling in normal and pathological states [44]. Changes in the extracellular matrix are involved in the pathogenesis of burn wounds. In particular, MMPs mediate such biological processes as inflammation, tissue remodeling, and angiogenesis. As a rule, the level of MPPs increases in the wounds since they are necessary to destroy the wound lodge, contributing to the healing of the wounds and the formation of scars [44].

It has been shown that MMPs levels were higher in non-healing wounds compared to well-healing wounds [45]. A significant increase in MMP-2 was noted from 3 to 21 days after the burn [46], and increased ProMMP-1, MMP-3, and MMP-9 levels were pointed out for the first three weeks after the severe burn injury [47].

Dysregulation of MMPs led to prolonged inflammation and delayed wound healing. Therefore, MMPs are essential biomarkers for assessing wound healing in burns.

### 4.5. Reactive Oxygen Species (ROS)

Many processes associated with the oxidation of biological molecules are accompanied by reactive oxygen species (ROS) generation. ROS, which is formed in the area of inflammation, includes free radicals, in particular, superoxidanion radical (O2^−^˙), hydroxyl radical (OH˙), hydrogen peroxide (H_2_O_2_), singlet oxygen (‘O2), etc.

The response to a burn injury depends on the balance between free radical production and detoxification. On the one hand, free radicals produced by activated neutrophils have an antimicrobial effect and a beneficial effect on wound healing. On the other hand, excessive formation of ROS and products of lipid peroxidation (LPO) in severe burn injury is accompanied by oxidative stress and is associated with the development of systemic inflammatory response, immunosuppression, promotion of the development of bacteremia and sepsis, and systemic tissue damage [48,49].

ROS and LPO slow down tissue regeneration processes and reduce the viability of fibroblasts and keratinocytes, which can be regarded as a sign of unfavorable wound healing. The uncontrolled formation of ROS by neutrophils and macrophages occurs in the early stages of the wound process and the acute period of burn disease [48]. Several authors note increased ROS production in the bloodstream during severe burn injury [50].

### 4.6. Nitric Oxide (NO)

As a biomarker, the wound pressure is indicative of the level of nitrogen oxide. Nitric oxide (NO) is an essential prognostic biomarker in assessing the severity of burn injury and wound healing. NO takes part in inflammation mechanisms, performing both proinflammatory and anti-inflammatory functions. The authors consider NO an essential factor in immunological reactivity necessary for implementing cytoprotective regulatory processes at the level of the cell and the whole macroorganism involved in antimicrobial protection [51,52].

Numerous experimental and clinical studies testify to changes in the activity of NO generation and its function during thermal injury. The authors believe that the deprivation of NO activity leads to disruption of the healing process. The integrated monitoring of NO, MMP, and the level of bacterial load will help accelerate the healing of chronic wounds [19,53].

Characterizing the hemostasis system as a whole, the authors note that in the first 48 h in patients with thermal injury, the procoagulant potential increases, and the parameters of the fibrinolytic cascade indicate both hyper- and hypofibrinolysis [7].

### 4.7. Parameters of the Hemostasis System

Assessment of the risk of coagulopathy and DIC syndrome in patients with a burn injury is the most crucial diagnostic purpose in combustiology. Severe burns (with a TBSA of more than 30%) are associated with severe coagulopathy [7]. Signs of coagulopathy have been noted on admission to the hospitalization in 39.3% of patients with severe burns [54]. In five weeks after burns injury, R. H. Bartlett et al. observed coagulopathy in patients with extensive burns (with a TBSA of 30–68%) [55].

The most important indicator of the development of coagulopathy is a significant increase in the level of D-dimer and fibrin cleavage products [56]. Numerous reports have shown that D-dimer levels in burn patients are significantly elevated on admission to hospital [57,58,59].

Disorders in the fibrinolysis system are also observed in proportion to the injury severity in burn patients [59,60].

Characterizing the hemostasis system as a whole, the authors note that in the first 48 h in patients with thermal injury, the procoagulant potential increases, and the parameters of the fibrinolytic cascade indicate both hyper- and hypo-fibrinolysis [7].

Thus, assessing the risk of developing coagulopathy and DIC syndrome in patients with severe thermal injury is the most crucial diagnostic task in combustiology.

## 5. Conclusions

Thus, numerous biologically active substances related to inflammatory mediators are involved in the immunopathogenesis of the development of local and systemic disorders in burn injury. Cytokines, growth factors, C-reactive protein, procalcitonin and presepsin, matrix metalloproteases, reactive oxygen species, nitric oxide, structural proteins [70] and hemostasis parameters have the most significant importance in burn injury. These immunobiochemical markers are considered prognostic biomarkers in the assessment of the severity of burn injury and wound healing and, ultimately, in the personalized therapy of burn patients.

Studies on the search for new biomarker candidates and methods for their detection allow not only the prediction of wound healing but also open new potential targets for therapy in burns. However, additional studies are required to develop clinically significant diagnostic tests to understand the biological reaction to a burn injury.

## Figures and Tables

**Table 1:** Immunobiochemical markers of severe burn injury and burn sepsis.

Markers	Biomarker Level (Parameter)	Ref.
Cytokines (proinflammatory IL-1α, IL-6, TNFα)	Levels of proinflammatory cytokines (IL-1β, IL-6, and TNF-α) increase within 24–48 h after a burn Levels of anti-inflammatory cytokines (IL-1 receptor antagonist, IL-4, IL-10, IL-11, and IL-13) are reduced (in cases with sepsis levels that are significantly higher than cases without sepsis)	[27,30]
C-reactive protein (CRP)	Levels increased in cases with sepsis	[34,35,36]
Procalcitonin (PCT) and presepsin	Levels increased in cases with sepsis	[32,38,39,40,41,42,43]
Growth factors (IGFBR-1, IGFBR-3, YGF, bFGF, IGF-1, TGF)	Levels increased	[30,31,32,33]
Matrix metalloproteinases (MMP-1, 2, 3, 9, etc.)	Activity increased	[44,45,46,47,48]
Reactive oxygen species (ROS)	Levels increased due to oxidative stress	[49,50,51,52]
Nitric oxide (NO)	Levels increased	[19,53]
Parameters of the hemostasis system (platelets, fibrinogen, D-dimer, protamine sulfate, fibrin degradation products, activated partial thromboplastin time, prothrombin time, and thrombin time)	Abnormal coagulation parameters: - thrombocytopenia 24–48 h after burn; - hypercoagulability and was attributed to high levels of fibrinogen and thromboplastin due to tissue lysis; Signs of coagulopathy and DIC syndrome	[54,55,56,57,58,59,60,61]
Inflammatory markers (IL-1β, Il-6, IL-8, TNFα, INFχ, IL12h70, Il-17, Il 2, Il-4, IL-5, IL-10, IL-13, IL-7	Levels increased	[61,62]
Stress marker (adrenaline, noradrenaline, dopamine, cortisol)	Levels increased	[63,64,65]
Hormones (leptin, progesterone, insulin, thyroid hormones)	Levels increased; levels of thyroid hormones lowered	[61,66]
Structural proteins (proteasome, type IV collagen, lfminin-5, pyrodinoline, deoxypyrodinoline)	Levels vary depending on the severity of the burn injury and sepsis	[67,68,69]

## References

[1] National Burn Repository (2019). Update, Report of Data from 2009–2018. American Burn Assotiation NBR Advisory Committee. Dataset Version 14.0. Ameriburn.siteym.com.2019. https://www.who.int/publications/i/item/9789240011311.

[2] Zhang P., Zou B., Liou Y.C., Huang C. (2021). The pathogenesis and diagnosis of sepsis post burn injury. Burn. Trauma.

[3] Nielson C.B., Duethman N.C., Howard J.M., Moncure M., Wood J.G. (2017). Burns: Pathophysiology of Systemic Complications and Current Management. J. Burn Care Res..

[4] Porter C., Herndon D.N., Bhattarai N., Ogunbileje J.O., Szczesny B., Szabo C., Toliver-Kinsky T., Sidossis L.S. (2016). Differential acute and chronic effects of burn trauma on murine skeletal muscle bioenergetics. Burns.

[5] Singer M., Deutschman C.S., Seymour C.W., Shankar-Hari M., Annane D., Bauer M., Bellomo R., Bernard G.R., Chiche J.D., Coopersmith C.M. (2016). The Third International Consensus Definitions for Sepsis and Septic Shock (Sepsis-3). JAMA.

[6] Greenhalgh D.G. (2017). Sepsis in the burn patient: A different problem than sepsis in the general population. Burn. Trauma.

[7] Hotchkiss R.S., Moldawer L.L., Opal S.M., Reinhart K., Turnbull I.R., Vincent J.L. (2016). Sepsis and septic shock. Nat. Rev. Dis. Primers.

[8] Ball R.L., Keyloun J.W., Brummel-Ziedins K., Orfeo T., Palmieri T.L., Johnson L.S., Moffatt L.T., Pusateri A.E., Shupp J.W. (2020). Burn-Induced Coagulopathies: A Comprehensive Review. Shock.

[9] Sherren P.B., Hussey J., Martin R., Kundishora T., Parker M., Emerson B. (2013). Acute burn induced coagulopathy. Burns.

[10] Evers L.H., Bhavsar D., Mailänder P. (2010). The biology of burn injury. Exp. Dermatol..

[11] Ravat F., Payre J., Peslages P., Fontaine M., Sens N. (2011). Burn: An inflammatory process. Pathol. Biol..

[12] Peng D.Z. (2008). Researches in immunological responses after burn injury in China. Chin. J. Burn..

[13] Moins-Teisserenc H., Cordeiro D.J., Audigier V., Ressaire Q., Benyamina M., Lambert J., Maki G., Homyrda L., Toubert A., Legrand M. (2021). Severe altered immune status after burn injury is associated with bacterial infection and septic shock. Front. Immunol..

[14] Porter C., Tompkins R.G., Finnerty C.C., Sidossis L.S., Suman O.E., Herndon D.N. (2016). The metabolic stress response to burn trauma: Current understanding and therapies. Lancet.

[15] Schwacha M.G. (2003). Macrophages and post-burn immune dysfunction. Burns.

[16] Gosain A., Gamelli R.L. (2005). A primer in cytokines. J. Burn Care Rehabil..

[17] Schwacha M.G., Ayala A., Chaudry I.H. (2000). Insights into the role of γδ T lymphocytes in the immunopathogenic response to thermal injury. J. Leukoc. Biol..

[18] Kim A., Lang T., Xue M., Wijewardana A., Jackson C., Vandervord J. (2017). The Role of Th-17 Cells and γδ T-Cells in Modulating the Systemic Inflammatory Response to Severe Burn Injury. Int. J. Mol. Sci..

[19] Luo G., Peng D., Zheng J., Chen X., Wu J., Elster E.A., Tadaki D.K. (2005). The role of NO in macrophage dysfunction at early stage after burn injury. Burns.

[20] Laggner M., Lingitz M.T., Copic D., Direder M., Klas K., Bormann D., Gugerell A., Moser B., Radtke C., Hacker S. (2022). Severity of thermal burn injury is associated with systemic neutrophil activation. Sci. Rep..

[21] Rani M., Zhang Q., Schwacha M.G. (2014). Burn wound γδ T-cells support a Th2 and Th17 immune response. J. Burn Care Res..

[22] Hanschen M., Tajima G., O’Leary F., Ikeda K., Lederer J.A. (2011). Injury induces early activation of T-cell receptor signaling pathways in CD4+ regulatory T cells. Shock.

[23] Huang L.F., Yao Y.M., Dong N., Yu Y., He L., Sheng Z. (2010). Association between regulatory T cell activity and sepsis and outcome of severely burned patients: A prospective, observational study. Crit. Care.

[24] Carlton M., Voisey J., Parker T.J., Punyadeera C., Cuttle L. (2021). A review of potential biomarkers for assessing physical and psychological trauma in paediatric burns. Burn. Trauma.

[25] Boldeanu L., Boldeanu M.V., Bogdan M., Meca A.D., Coman C.G., Buca B.R., Tartau C.G., Tartau L.M. (2020). Immunological approaches and therapy in burns (Review). Exp. Ther. Med..

[26] Mohd J., Shah Y., Omar E., Pai D.R., Sood S. (2012). Cellular events and biomarkers of wound healing. Indian J. Plast. Surg..

[27] Harding K.G., Morris H.L., Patel G.K. (2002). Science, medicine and the future: Healing chronic wounds. BMJ.

[28] Vinaik R., Abdullahi A., Barayan D., Jeschke M.G. (2020). NLRP3 inflammasome activity is required for wound healing after burns. Transl. Res..

[29] Finnerty C.C., Herndon D.N., Chinkes D.L., Jeschke M.G. (2007). Serum cytokine differences in severely burned children with and without sepsis. Shock.

[30] Jeschke M.G., Gauglitz G.G., Kulp G.A., Finnerty C.C., Williams F.N., Kraft R., Suman O.E., Mlcak R.P., Herndon D.N. (2011). Long-term persistence of the pathophysiologic response to severe burn injury. PLoS ONE.

[31] Jeschke M.G., Barrow R.E., Herndon D.N. (2004). Extended hypermetabolic response of the liver in severely burned pediatric patients. Arch. Surg..

[32] Abdel-Hafez N.M., Saleh Hassan Y., El-Metwally T.H. (2007). A study on biomarkers, cytokines, and growth factors in children with burn injuries. Ann. Burn. Fire Disasters.

[33] Sherbet G.V. (2011). The epidermal growth factor (EGF) family. Growth Factors and Their Receptors in Cell Differentiation, Cancer and Cancer Therapy.

[34] Jeschke M.G., Finnerty C.C., Kulp G.A., Kraft R., Herndon D.N. (2013). Can we use C-reactive protein levels to predict severe infection or sepsis in severely burned patients?. Int. J. Burn. Trauma.

[35] Joby J., Meer M., Krishnakumar G. (2017). C-reactive protein: An early predictor of sepsis in patients with thermal burns. Int. Surg. J..

[36] Lee H.Y., Kaneki M., Andreas J., Tompkins R.G., Martyn J.A. (2011). Novel mitochondria-targeted antioxidant peptide ameliorates burn-induced apoptosis and endoplasmic reticulum stress in the skeletal muscle of mice. Shock.

[37] Cabral L., Afreixo V., Almeida L., Paiva J.A. (2016). The Use of Procalcitonin (PCT) for Diagnosis of Sepsis in Burn Patients: A Meta-Analysis. PLoS ONE.

[38] Cakir M.Ö., Yakupoglu S.B.N., Yücel N., Akbaba O.K.D. (2014). Evaluation of soluble CD14 subtype (presepsin) in burn sepsis. Burns.

[39] Lavrentieva A., Kontakiotis T., Lazaridis L., Tsotsolis N., Koumis J., Kyriazis G., Bitzani M. (2007). Inflammatory markers in patients with severe burn injury: What is the best indicator of sepsis?. Burns.

[40] Mokline A., Garsallah L., Rahmani I., Jerbi K., Oueslati H., Tlaili S., Hammouda R., Gasri B., Messadi A.A. (2015). Procalcitonin: A diagnostic and prognostic biomarker of sepsis in burned patients. Ann. Burn. Fire Disasters.

[41] Velissaris D., Zareifopoulos N., Karamouzos V., Karanikolas E., Pierrakos C., Koniari I., Karanikolas M. (2021). Presepsin as a Diagnostic and Prognostic Biomarker in Sepsis. Cureus.

[42] Masson S., Caironi P., Spanuth E., Thomae R., Panigada M., Sangiorgi G., Fumagalli R., Mauri T., Isgrò S., Fanizza C. (2014). Presepsin (soluble CD14 subtype) and procalcitonin levels for mortality prediction in sepsis: Data from the Albumin Italian Outcome Sepsis trial. Crit. Care.

[43] Egea-Guerrero J.J., Martínez-Fernández C., Rodríguez-Rodríguez A., Bohórquez-López A., Vilches-Arenas A., Pacheco-Sánchez M., Guerrero J.M., Murillo-Cabezas F. (2015). The utility of C-reactive protein and procalcitonin for sepsis diagnosis in critically burned patients: A preliminary study. Plast. Surg..

[44] Laronha H., Caldeira J. (2020). Structure and function of human matrix metalloproteinases. Cells.

[45] Utz E.R., Elster E.A., Tadaki D.K., Gage F., Perdue P.W., Forsberg J.A., Stojadinovic A., Hawksworth J.S., Brown T.S. (2010). Metalloproteinase expression is associated with traumatic wound failure. J. Surg. Res..

[46] Weremijewicz A., Matuszczak E., Sankiewicz A., Tylicka M., Komarowska M., Tokarzewicz A. (2018). Matrix metalloproteinase-2 and its correlation with basal membrane components laminin-5 and collagen type IV in paediatric burn patients measured with surface Plasmon resonance imaging (SPRI) biosensors. Burns.

[47] Dasu M.R., Spies M., Barrow R.E., Herndon D.N. (2003). Matrix metalloproteinases and their tissue inhibitors in severely burned children. Wound Repair Regen..

[48] Parihar A., Parihar M.S., Milner S., Bhat S. (2008). Oxidative stress and anti-oxidative mobilization in burn injury. Burns.

[49] Horton J.W. (2003). Free radicals and lipid peroxidation mediated injury in burn trauma: The role of antioxidant therapy. Toxicology.

[50] Auger C., Samadi O., Jeschke M.G. (2017). The biochemical alterations underlying post-burn hypermetabolism. Biochim. Biophys. Acta Mol. Basis Dis..

[51] Lestaevel P., Agay D., Peinnequin A., Cruz C., Cespuglio R., Clarençon D., Multon E., Chancerelle Y. (2003). Effects of a thermal injury on brain and blood nitric oxide (NO) content in the rat. Burns.

[52] Haycock J.W., Ralston D.R., Morris B., Freedlander E., MacNeil S. (1997). Oxidative damage to protein and alterations to antioxidant levels in human cutaneous thermal injury. Burns.

[53] Privett B.J., Shinb J.H., Schoenfisch M.H. (2010). Tutorial Review: Electrochemical Nitric Oxide Sensors for Physiological Measurements. Chem. Soc. Rev..

[54] Glas G.J., Levi M., Schultz M.J. (2016). Coagulopathy and its management in patients with severe burns. J. Thromb. Haemost..

[55] Bartlett R.H., Fong S.W., Marrujo G., Hardeman J., Anderson W. (1981). Coagulation and platelet changes after thermal injury in man. Burns.

[56] Hayakawa M., Maekawa K., Kushimoto S., Kato H., Sasaki J., Ogura H., Matauoka T., Uejima T., Morimura N., Ishikura H. (2016). High D-Dimer levels predict a poor outcome in patients with severe trauma, even with high fibrinogen levels on arrival: A multicenter retrospective study. Shock.

[57] Kowal-Vern A., Walenga J.M., McGill V., Gamelli R.L. (2001). The impact of antithrombin (H) concentrate infusions on pulmonary function in the acute phase of thermal injury. Burns.

[58] King D.R., Namias N., Andrews D.M. (2010). Coagulation abnormalities following thermal injury. Blood Coagul. Fibrinolysis.

[59] Lavrentieva A., Kontakiotis T., Bitzani M., Papaioannou-Gaki G., Parlapani A., Thomareis O., Tsotsolis N., Giala M.A. (2008). Early coagulation disorders after severe burn injury: Impact on mortality. Intensive Care Med..

[60] Garcia-Avello A., Lorente J.A., Cesar-Perez J., Garcia-Frade L.J., Alvarado R., Arevalo J.M., Navarro J.L., Esteban A. (1998). Degree of hypercoagulability and hyperfibrinolysis is related to organ failure and prognosis after burn trauma. Thromb. Res..

[61] Lu R.P., Ni A., Lin F.C., Ortiz-Pujols S.M., Adams S.D., Monroe D.M., Whinna H.C., Cairns B.A., Key N.S. (2013). Major burn injury is not associated with acute traumatic coagulopathy. J. Trauma Acute Care Surg..

[62] Kraft R., Herndon D.N., Finnerty C.C., Cox R.A., Song J., Jeschke M.G. (2015). Predictive Value of IL-8 for Sepsis and Severe Infections After Burn Injury: A Clinical Study. Shock.

[63] Chester S.J., Stockton K., De Young A., Kipping B., Tyack Z., Griffin B., Chester R.L., Kimble R.M. (2016). Effectiveness of medical hypnosis for pain reduction and faster wound healing in pediatric acute burn injury: Study protocol for a randomized controlled trial. Trials.

[64] Jeschke M.G., Finnerty C.C., Herndon D.N., Song J., Boehning D., Tompkins R.G., Baker H.V., Gauglitz G.G. (2012). Severe injury is associated with insulin resistance, endoplasmic reticulum stress response, and unfolded protein response. Ann. Surg..

[65] Kraft R., Kulp G.A., Herndon D.N., Emdad F., Williams F.N., Hawkins H.K., Leonard K.R., Jeschke M.G. (2011). Is there a difference in clinical outcomes, inflammation, and hypermetabolism between scald and flame burn?. Pediatr. Crit. Care Med..

[66] Zang T., Broszczak D.A., Broadbent J.A., Cuttle L., Lu H., Parker T.J. (2016). The biochemistry of blister fluid from pediatric burn injuries: Proteomics and metabolomics aspects. Expert Rev. Proteomics..

[67] Lavrentieva A., Voutsas V., Konoglou M., Karali V., Koukiasa P., Loridas N., Papaioannou M., Vasileiadou G., Bitzani M. (2017). Determinants of Outcome in Burn ICU Patients with Septic Shock. J. Burn Care Res..

[68] Matwiyoff G.N., Prahl J.D., Miller R.J., Carmichael J.J., Amundson D.E., Seda G., Daheshia M. (2012). Immune regulation of procalcitonin: A biomarker and mediator of infection. Inflamm. Res..

[69] Tan J., Wu J. (2017). Current progress in understanding the molecular pathogenesis of burn scar contracture. Burn. Trauma.

[70] Sierawska O., Małkowska P., Taskin C., Hrynkiewicz R., Mertowska P., Grywalska E., Korzeniowski T., Torres K., Surowiecka A., Niedźwiedzka-Rystwej P. (2022). Innate Immune System Response to Burn Damage-Focus on Cytokine Alteration. Int. J. Mol. Sci..

